# Association between home entrance characteristics and depression: A cross-sectional study of community-dwelling older adults in Japan

**DOI:** 10.1016/j.pmedr.2025.103148

**Published:** 2025-06-20

**Authors:** Hiroaki Yoshida, Masamichi Hanazato, Yoko Matsuoka, Yu-Ru Chen, Aiko Eguchi, Yusuke Mizuno, Hiroki Suzuki

**Affiliations:** a Center for Preventive Medical Sciences, Chiba University, 1-33, Yayoicho, Inage-ku, Chiba, Chiba 263-8522, Japan; b Graduate School of Science and Engineering, Chiba University, 1-33, Yayoicho, Inage-ku, Chiba, Chiba 263-8522, Japan; c Design Research Institute, Chiba University, 1-19-1, Bunka, Sumida-ku, Tokyo 131-0044, Japan

**Keywords:** Entrance porch, Depression, Mental health, Built environment, Older adults, Front porch

## Abstract

**Objective:**

Depression among older adults is associated with cognitive function decline and increased risk of premature mortality. Numerous studies have reported associations between residential environments and depression. However, few have investigated the association between home entrance characteristics and depression. Therefore, this study aims to clarify the association between entrance characteristics and depression among older adults.

**Methods:**

A cohort study was conducted among Japanese adults aged 65 years and older using data collected in January 2022 and October 2023. The analysis included 2046 individuals (mean age: 74.8 ± 6.2 years) in one ward in Tokyo. Depression in 2023 was assessed using the Geriatric Depression Scale-15. Explanatory variables were entrance area characteristics in 2023. Modified Poisson regression was performed to estimate prevalence ratios and 95 % confidence intervals (CI).

**Results:**

Of the 2046 participants, 458 (22.4 %) were classified as depressed. Compared with participants living in homes without plants or flowers near the entrance, those living with plants were associated with lower prevalence of depression (prevalence ratio: 0.84; 95 % CI: 0.71–0.98). Stratified analysis by housing type showed that in apartment buildings, prevalence ratio of depression among those with plants or flowers was 0.72 (95 % CI: 0.52–0.99). However, no significant association was observed among residents of detached houses (prevalence ratio: 0.85; 95 % CI: 0.70–1.03).

**Conclusion:**

Our findings underscore the importance of considering residential entrance features to support the mental health of older adults. Entrance designs and management systems that allow the placement of plants and flowers can help reduce depression among older adults.

## Highlights

• Association between house entrance features and depression in older adults examined.
• Overall and housing-type stratified analyses conducted.
• Homes with entrance plants were associated with 16 % lower prevalence of depression.
• For apartments, entrance plants were associated with 28 % lower prevalence of depression.
• Findings can guide housing design to support older adults' mental health.

## 1 Introduction

Depression is among the most common diseases worldwide, and a serious public health problem. While prevalence varies by country and region, the global prevalence in 2015 was 4.4 %, with higher rates observed in developed countries (World Health Organization, 2017). Approximately 14 % of adults aged 60 years and older worldwide are reported to suffer from mental disorders (World Health Organization, 2023b) including 5.7 % from depression (Institute of Health Metrics and Evaluation, 2019; World Health Organization, 2023a). Mental disorders among older adults account for 10.6 % of disability-adjusted life years in this age group (World Health Organization, 2023b). Limitations in mobility and physical function associated with aging, as well as life events such as retirement, reduced social interactions, or the loss of a spouse, are associated with an increased risk of depression among older adults (Chao, 2014; Fiske et al., 2009). Poor mental health among middle-aged and older adults has been associated with an increased risk of dementia (Holmquist et al., 2020) and mortality (Zhang et al., 2023). Depression among older adults is associated with declines in physical and cognitive function and an increased risk of premature mortality (Fiske et al., 2009). Promoting and maintaining mental health is essential for a healthy and quality later life.

Numerous studies have reported associations between neighborhood environments, indoor housing environments, and mental health. For example, older adults who perceive their neighborhoods as safe or experience a strong sense of social cohesion have a lower risk of depression (Choi and Matz-Costa, 2017). Additionally, the presence of more green space in residential areas has been associated with a lower risk of depression among older adults (Nishigaki et al., 2020). Regarding the residential environments, studies of people aged 60 years and older have reported associations between the risk of depression and factors such as smaller dwellings (Cheung, 2024; Qiu et al., 2020), insufficient number of rooms (Firdaus, 2017), and inadequate living facilities (Firdaus, 2017). These findings suggest that creating and maintaining an appropriate residential environment can significantly improve older adults' mental health. In particular, older adults tend to spend more time at home than other age groups (Spalt et al., 2016), making the impact of the home environment on their mental health more significant.

While numerous studies have reported associations between mental health and both the neighborhood environment and indoor housing environments, research focusing on the intermediate spaces between these two, such as the areas around home entrances, remains limited. Front yard elements, such as seating, porches, and flowerpots, were reported to be associated with a stronger sense of place among residents in a neighborhood of Buffalo, New York (Kickert et al., 2024). It has been reported that older adults living in neighborhoods with homes that have stoops or covered front porches tend to have higher satisfaction with social support and maintain or improve physical functions such as walking speed and grip strength (Brown et al., 2008). Front porches have been suggested to play a role in strengthening connections between residents and their neighbors (Brown et al., 2008; Cabrera and Najarian, 2015; Chalmin-Pui et al., 2023). Incorporating plants into front gardens reduced stress and improved cortisol levels in a study of 38 homes in northern England (Chalmin-Pui et al., 2021). In Tokyo, potted plants facing the street are associated with more frequent neighbor interactions (Fujitani and Kobayashi, 2017). These findings suggest that entrance area characteristics may enhance neighborhood attachment and social interactions, potentially promoting better mental health. However, no studies have examined the association between the characteristics of the entrance area and mental health. Therefore, this study aims to examine whether home entrance characteristics are associated with depression among older adults. Detached houses typically have a dedicated space between the entrance and the road. In contrast, entrances in apartment buildings are connected to shared corridors, resulting in significant differences in how the entrance area is utilized. Given the substantial differences in entrance-area characteristics between detached houses and apartment buildings, this study also seeks to determine whether entrance area characteristics associated with depression differ between these housing types. The results of this study provide new insights into housing environment research by examining the “intermediate area” between the neighborhood and the housing environment, specifically the entrance area. These findings could serve as evidence to guide housing design aimed at promoting the mental health of older adults.

## 2 Methods

### 2.1 Study design and participants

**Fig. 1:**
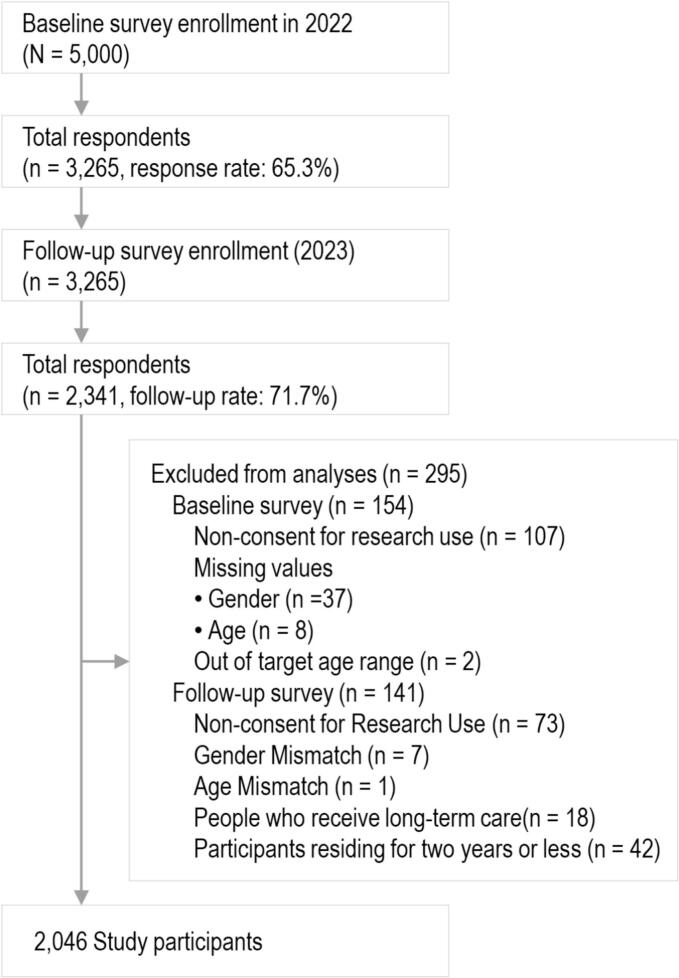
Flow chart of selection of older adults for study participation in Japan (2022−2023).

We used longitudinal cohort data collected through self-administered questionnaires in January 2022 (baseline) and October 2023 (follow-up) in one ward, an urban residential area located in the eastern part of Tokyo, and characterized by a high population density that exceeds the average of Tokyo's 23 wards. The area features a mixed land use of residential, commercial, and industrial zones, with a regional identity rooted in manufacturing, while the tertiary sector dominates its urban industrial structure. Regarding residential characteristics, the area includes densely packed wooden houses that survived wartime destruction (Fig. S1A), as well as redeveloped zones of mid- to high-rise condominiums and detached homes, resulting in a variety of entrance characteristics (Fig. S1B). In the baseline survey, self-administered questionnaires were mailed to 5000 people aged ≥65 years who were physically and cognitively independent, not eligible for public long-term care insurance, and living independently in the community. The survey was conducted using the random sampling method. In the follow-up survey, self-administered questionnaires were mailed to the 3265 individuals who participated in the baseline survey, and 2341 participants responded, resulting in a follow-up rate of 71.7 %. Of the 2341 respondents, 154 individuals were excluded because they did not consent to participate at baseline, had missing gender or age data, or were under 65 years of age at baseline. At follow-up, an additional 141 individuals were excluded because they did not consent to participate, had inconsistencies in reported gender or age, had a care level of 2 or higher under the long-term care insurance system, or had resided in their current home for approximately two years or less to exclude those who were likely to have moved during the study period, as the influence of their previous residential environment may still have been present. Ultimately, 2046 individuals (including 1141 women) were included in the analysis (Fig. 1). All participants were provided with a document explaining the purpose and outline of the study, and a consent form was included with the questionnaire. Consent to participate in the study was obtained through the consent form. Ethical approval for the study was obtained from the Ethics Committee at Chiba University (Approval number: M10451).

### 2.2 Variable

#### 2.2.1 Depressive symptoms

The outcome variable was depressive symptoms in 2023. Depressive symptoms were evaluated in both the baseline survey in 2022 and the follow-up survey in 2023, and assessed with the Japanese short version of the Geriatric Depression Scale (GDS) (Niino, 1991; Sugishita et al., 2017; Yesavage et al., 1982). Participants were classified into two groups: without depressive symptoms (GDS < 5) and with depressive symptoms (GDS ≥ 5) (Brink et al., 1982; Nyunt et al., 2009).

#### 2.2.2 Explanatory variables

The explanatory variables were defined as the presence or absence of the following seven features around the entrance area in 2023: stairs, eaves or overhangs, plants or flowers, parking, benches or seating outside the entrance, sliding entrance doors, and an earthen floor area inside the entrance. Based on features identified in previous studies (Brown et al., 2008), features that may promote social interaction and physical activity, both of which are associated with mental health (Misawa and Kondo, 2019; Pearce et al., 2022), were selected as explanatory variables. Stairs, eaves or overhangs, and benches or seats-key components of porch spaces that have been suggested to facilitate social support (Brown et al., 2008), were included as explanatory variables. Parking areas near entrances often serve as storage areas for car-related items, gardening tools, and outdoor equipment. These spaces may encourage activities such as car maintenance, cleaning around the entrance, and gardening, which may promote physical activity. Therefore, parking spaces were included as an explanatory variable. Sliding doors were included as an explanatory variable because they efficiently utilize the space around the entrance and allow the occupants to freely adjust the degree of opening, providing a flexible connection to the street. Indoor entrance area with earthen flooring, an intermediate space between the exterior and interior, is thought to facilitate casual social interactions. Both sliding doors and indoor entrance areas with earthen flooring are unique features of Japanese residences, serving as transitional elements between outdoor and indoor spaces. These features may promote interactions between residents and their neighbors and were therefore included as explanatory variables.

#### 2.2.3 Covariates

Based on the literature (Cheung, 2024; Nishigaki et al., 2020; Qiu et al., 2020), the following sociodemographic and health factors were adjusted in regressions as covariates: gender (men, women, others), age group (65–74 or ≥ 75), years of education (< 10 or ≥ 10 years), annual equivalent income, social isolation, employment status (not employed, employed), instrumental activities of daily-living (IADL), frequency of going out (< 1 day or ≥ 1 day per week), self-reported chronic diseases, and population density of the residential area. Annual equivalent income was calculated by dividing household income by the square root of household size and categorized into <2.0, 2.0–3.9, or ≥ 4.0 million yen. We included a social isolation scale comprising three items: living alone, less than monthly contact with friends, and no weekly participation in social activities (e.g., sports, hobbies, and volunteering). Scores range from 0 to 3, with ≥2 indicating social isolation (Nakagomi et al., 2024; Wang et al., 2023). IADL were assessed using the Tokyo Metropolitan Institute of Gerontology Index of Competence (Koyano et al., 1991), and the results were classified as good (5 points) or poor (≤ 4 points). We adjusted for housing type (detached house or apartment building) in the analysis for all participants, as the entrance environment differs considerably between detached houses and apartment buildings. All covariates were derived from the baseline survey conducted in 2022, 1 year and 9 months before the follow-up survey. Additionally, depression in 2022 was adjusted in the analysis to partially account for reverse causation (Vanderweele et al., 2020), where the depression score in 2022 could influence improvements or changes to the characteristics of the entrance area, such as planting greenery, replacing the door, or upgrading the parking lot, in 2023, or could influence depression in 2023. We did not control for loneliness, as it is both a symptom and a risk factor for depression (Lee et al., 2021; (Van As et al., 2022)); thus, including loneliness as a covariate could result in over-control.

### 2.3 Statistical analysis

Multiple imputation by chained equations was used to address potential bias owing to missing data. Following previous research (Sterne et al., 2009), all variables included in the analysis, such as the outcome variables, explanatory variables, and covariates, were imputed. Table 1 presents the number of participants for whom data was imputed. Assuming that the missing data were missing at random, 20 imputed records were generated using the chained equation procedure (White et al., 2011). To examine the association between entrance area characteristics and depression, modified Poisson regression analysis was used to calculate prevalence ratios and 95 % confidence intervals (CI) for all participants. This approach was chosen because the prevalence of depression in 2023 was relatively high (>10 %), and odds ratio obtained from logistic regression models may substantially overestimate prevalence ratios under such conditions (Barros and Hirakata, 2003; McNutt et al., 2003). Additionally, modified Poisson regression analyses stratified by housing type were used to assess differences in the effect of entrance area characteristics on depression between detached houses and apartment buildings. All explanatory variables were entered simultaneously in the models. All statistical analyses and multiple imputations were performed using Stata/SE version 15.1 (StataCorp LLC, College Station, TX, USA) with statistical significance inferred at a two-tailed *p*-value of <0.05.

**Table 1:** Descriptive statistics of variables among older adults in Japan (2022–2023).

		Total		GDS score in 2023					
				Non-depressed (<5)		Depression (≥5)		Missing	
		n	%	N	%	n	%	n	%
GDS score in 2023		2046	100.0	1185	57.9	458	22.4	403	19.7
Stairs from the road									
	Presence	453	22.1	271	22.9	95	20.7	87	21.6
Eaves or overhangs to avoid rain									
	Presence	905	44.2	572	48.3	186	40.6	147	36.5
Indoor entrance area with earthen flooring									
	Presence	483	23.6	297	25.1	104	22.7	82	20.3
Parking lot									
	Presence	454	22.2	291	24.6	82	17.9	81	20.1
Sliding entrance door									
	Presence	246	12.0	147	12.4	50	10.9	49	12.2
Bench or seating inside the entrance									
	Presence	112	5.5	71	6.0	18	3.9	23	5.7
Bench or seating outside the entrance									
	Presence	41	2.0	27	2.3	8	1.7	6	1.5
Plants or flowers									
	Presence	790	38.6	490	41.4	147	32.1	153	38.0
Gender									
	Men	905	44.2	555	46.8	221	48.3	129	32.0
	Women	1141	55.8	630	53.2	237	51.7	274	68.0
Age groups (years)									
	65–74	1088	53.2	685	57.8	240	52.4	163	40.4
	≥75	958	46.8	500	42.2	218	47.6	240	59.6
Education (years)									
	<10	401	19.6	183	15.4	111	24.2	107	26.6
	≥10	1601	78.3	977	82.4	345	75.3	279	69.2
	Missing	44	2.2	25	2.1	2	0.4	17	4.2
Employment status									
	Not employed	1185	57.9	652	55.0	285	62.2	248	61.5
	Employed	785	38.4	500	42.2	152	33.2	133	33.0
	Missing	76	3.7	33	2.8	21	4.6	22	5.5
*Annual equivalent income (million yen)*									
	< 2.0	837	40.9	428	36.1	232	50.7	177	43.9
	2.0–3.9	670	32.7	419	35.4	135	29.5	116	28.8
	≥ 4.0	342	16.7	245	20.7	54	11.8	43	10.7
	Missing	197	9.6	93	7.8	37	8.1	67	16.6
Social isolation									
	Not isolated	837	40.9	546	46.1	126	27.5	165	40.9
	Isolated	1057	51.7	568	47.9	298	65.1	191	47.4
	Missing	152	7.4	71	6.0	34	7.4	47	11.7
Instrumental activities of daily-living									
	Good	1190	58.2	711	60.0	243	53.1	236	58.6
	Poor	780	38.1	444	37.5	194	42.4	142	35.2
	Missing	76	3.7	30	2.5	21	4.6	25	6.2
Frequency of going out									
	≥ 1/day	1944	95.0	1142	96.4	427	93.2	375	93.1
	< 1/day	62	3.0	23	1.9	21	4.6	18	4.5
	Missing	40	2.0	20	1.7	10	2.2	10	2.5
GDS score in 2022									
	Non-depressed (<5)	1183	57.8	920	77.6	102	22.3	161	40.0
	Depression (≥5)	487	23.8	123	10.4	285	62.2	79	19.6
	Missing	376	18.4	142	12.0	71	15.5	163	40.4
Disease Status									
	Presence	357	17.4	237	20.0	58	12.7	62	15.4
	Absence	1607	78.5	909	76.7	383	83.6	315	78.2
	Missing	82	4.0	39	3.3	17	3.7	26	6.5
Housing Type									
	Detached house	901	44.0	517	43.6	194	42.4	190	47.1
	Apartment building	1061	51.9	624	52.7	245	53.5	192	47.6
	Missing	84	4.1	44	3.7	19	4.1	21	5.2
*Population density (persons / km2 of inhabitable area)*									
	Mean (SD)	37,989 (7539)		38,339 (7537)		37,510 (7603)		37,512 (7432)	

GDS, Geriatric Depression Scale; SD, standard deviation.

## 3 Results

Of the 2046 participants (mean age: 74.8 years), 458 individuals (22.4 %) were classified as having depressive tendencies, while 403 individuals (19.7 %) had missing data in 2023. Table 2 presents the features of the front entrance area, while the following percentages of participants reported their presence: stairs (22.1 %), eaves or overhangs (44.2 %), indoor entrance area with earthen flooring (23.6 %), parking lot (22.2 %), sliding entrance doors (12.0 %), bench or seating inside the entrance (5.5 %), bench or seating outside the entrance (2.0 %), and plants or flowers (38.6 %). The most common feature was the presence of eaves or overhangs (44.2 %), while the least common was the presence of a bench or seating outside the entrance (2.0 %). Regarding the presence of entrance area features by housing type, a higher proportion of respondents living in detached houses reported the presence of all features compared with those living in apartment buildings. For example, 61.5 % of detached dwellers reported having plants or flowers compared with 18.4 % of apartment buildings.

**Table 2:** Features of the front entrance area of residences of older adults in Japan (2022–2023).

	Total		Housing Type					
			Detached house		Apartment building		Missing	
	*n* = 2046		*n* = 901		*n* = 1012		*n* = 133	
	N	%	N	%	n	%	n	%
Stairs from the road	453	22.1	233	25.9	199	19.7	21	15.8
Eaves or overhangs to avoid rain	905	44.2	443	49.2	422	41.7	40	30.1
Indoor entrance area with earthen flooring	483	23.6	280	31.1	181	17.9	22	16.5
Parking lot	454	22.2	302	33.5	127	12.5	25	18.8
Sliding entrance door	246	12.0	153	17.0	74	7.3	19	14.3
Bench or seating inside the entrance	112	5.5	66	7.3	41	4.1	5	3.8
Bench or seating outside the entrance	41	2.0	24	2.7	17	1.7	0	0.0
Plants or flowers	790	38.6	554	61.5	186	18.4	50	37.6

The prevalence ratio for depression was 0.84 (95 % CI: 0.71–0.98) among individuals with plants or flowers in the entrance area, while no significant associations were observed for other features (Table 3). Regarding the results of the modified Poisson regression analysis stratified by housing type, the prevalence ratio for depression among those with plants or flowers in apartment buildings was 0.72 (95 % CI: 0.52–0.99). However, no significant association was observed among detached house residents (prevalence ratio: 0.85; 95 % CI: 0.70–1.03).

**Table 3:** Prevalence ratios with 95 % confidence intervals for the association of depressive symptoms and the features of a front entrance among older adults in Japan (2022–2023).

	All participants	Housing Type	
	n = 2046	Detached house n = 901	Apartment building n = 1012
Front entrance features (ref: absence)	Prevalence ratio (95 % CI)	Prevalence ratio (95 % CI)	Prevalence ratio (95 % CI)
Stairs from the road	0.98 (0.83–1.15)	0.85 (0.67–1.09)	1.14 (0.94–1.39)
Eaves or overhangs to avoid rain	0.97 (0.85–1.10)	1.00 (0.80–1.24)	0.96 (0.79–1.16)
Indoor entrance area with earthen flooring	0.90 (0.76–1.07)	0.87 (0.71–1.05)	0.99 (0.79–1.26)
Parking lot	0.96 (0.78–1.18)	0.76 (0.38–1.50)	0.98 (0.67–1.43)
Sliding entrance door	0.96 (0.77–1.19)	0.66 (0.38–1.13)	1.16 (0.82–1.65)
Bench or seating inside the entrance	0.87 (0.60–1.27)	0.87 (0.68–1.11)	1.26 (0.71–2.25)
Bench or seating outside the entrance	0.98 (0.62–1.57)	0.91 (0.66–1.25)	1.01 (0.55–1.86)
Plants or flowers	0.84 (0.71–0.98)*	0.85 (0.70–1.03)	0.72 (0.52–0.99)*

CI: confidence interval; ref.: reference group. *p < 0.05. For “All participants,” adjusted for gender, age groups, employment status, education, annual equivalent income, social isolation, frequency of going out, depressive symptoms, housing type, and population density. For “Detached house” and “Apartment building,” adjustments excluded housing type, as it is inherent to the subgroup. The explanatory variables were included simultaneously, and all covariates are measured in 2022 (baseline). Depressive symptoms were assessed using the Japanese short version of the Geriatric Depression Scale; a score of ≥5 was used to classify participants as having depressive symptoms.

## 4 Discussion

To the best of our knowledge, this is the first study to examine the association between entrance features of homes and depression. In the analysis including all participants, we found that living in homes with plants or flowers near the entrance was associated with a lower prevalence of depression. In stratified analyses by housing type, the presence of plants or flowers around the entrance was associated with a lower prevalence of depression among apartment residents, whereas no such association was observed among residents of detached houses.

Previous studies show that front porches are beneficial for health (Brown et al., 2008). However, this study found no association between key components of porch spaces—such as stairs, eaves or overhangs, and benches or seats—and depression. In contrast, homes with plants or flowers around the entrance were associated with a lower prevalence of depression. It has been suggested that older adults living in areas with a higher prevalence of porches, compared with those in areas with fewer porches, benefit from increased social support, which may lead to reduced psychological distress (Brown et al., 2008). Front porches, defined as porches, balconies, or stoops with sufficient space to accommodate seating, provide areas for people to linger (Wilkerson et al., 2012), and have been suggested to help reinforce connections between residents and their neighbors (Brown et al., 2008; Cabrera and Najarian, 2015; Chalmin-Pui et al., 2023). Unlike front porches in Western countries, Japanese entrance porches are not typically designed as places to linger. They tend to consist of minimal areas with elements such as an awning for rain protection, potted plants, or an umbrella stand. In this study, only 2 % of the homes had a bench or seat outside the entrance. This finding suggests that houses with entrance porches designed for lingering, as seen in Western countries, are very limited in Japan. Such differences may have influenced the lack of association observed in this study between features such as benches or awnings and depression.

In such Japanese entrance porches, the presence of plants and flowers around the entrance may contribute to alleviating depression among residents, potentially through mechanisms related to social interactions during gardening activities. Qualitative studies show that front yard gardening naturally facilitates greetings and conversations between gardeners and passersby, thereby promoting social interactions within the community (Chalmin-Pui et al., 2023). Studies in Japanese residential areas have found that residents of homes with a higher number of potted plants facing the street interact more frequently with neighbors (Fujitani and Kobayashi, 2017), and plant care frequency has been positively linked to casual conversations (Mizukami, 2013). These findings suggest that placing plants near entrances, where encounters are likely to occur, may foster communication between neighbors. As social interaction and support are related to depressive symptoms (Lee et al., 2021), entrance greenery may help strengthen social ties, thereby reducing depression risk.

Additionally, although the effects of gardening beyond front entrances may also be involved, gardening activities may drive other mechanisms that positively affect mental health. The first mechanism is stress reduction through gardening activities. A meta-analysis conducted across all age groups has reported that gardening, including home gardening, is associated with lower stress levels and better mental health (Soga et al., 2017). Additionally, individuals who participate in home gardening have been found to experience reduced stress, increased happiness, and increased physical activity (Chalmin-Pui et al., 2021), suggesting that contact with nature through gardening may reduce stress and positively impact mental health. The second mechanism involves the influence of physical activity during gardening. Gardening has been reported to involve moderate-intensity physical activity (Bassett et al., 2000), and higher levels of physical activity have been associated with better mental health (Pearce et al., 2022). Therefore, individuals living in homes with plants or flowers may engage in increased physical activity through gardening, which could potentially contribute to better mental health.

The results of the stratified analysis by housing type showed that the association between having plants or flowers around the entrance area and a lower prevalence of depression remained significant only among residents of apartment buildings. Although a similar trend was observed among residents of detached houses, the association did not reach statistical significance, possibly owing to differences in the types of plants grown in detached houses and apartment buildings, or the high prevalence of plants or flowers in such homes.

In apartment buildings, where entrances are often connected to common corridors, plants are commonly grown in planters. Planter-grown plants usually require daily watering, resulting in nearly daily gardening activities. These activities, even if brief, provide opportunities to go outside, engage in light physical activity, interact with plants, and potentially foster social interactions, such as greeting neighbors or having casual conversations. In contrast, detached houses often provide space for plants to be grown directly in the ground. Ground-based vegetation, particularly garden trees, typically require minimal watering in Japan's climate, with maintenance limited to occasional pruning, often just once a year. Even herbaceous plants, such as flowering seedlings, generally require less frequent watering compared with those grown in planters. Daily gardening activities in the home may contribute to increased physical activity and enhanced social interactions, which may explain why the association between the presence of plants or flowers and reduced prevalence of depression remained significant only in apartment buildings.

In Japan, it is often prohibited to place personal belongings, including potted plants, in common areas of apartment buildings, making it difficult to keep plants or flowers near entrances. Nonetheless, 18.4 % of apartment building residents in this study reported having plants or flowers near their own entrance, suggesting that some apartment buildings may allow the placement of personal items, including plants or flowers (Fig. S2). By contrast, 61.5 % of residents of detached homes reported having plants or flowers around their entrance. This high prevalence in detached homes may explain the lack of significant differences based solely on the presence or absence of plants or flowers (Fig. S3). Future research should evaluate the quality of plants around entrances in detached homes, considering factors such as the number, plant type (e.g., groundcover plants, flowers, or trees and shrubs) and planting method (e.g., planting in planters or the ground).

This study is the first to examine the association between entrance-area environments and residents' mental health, providing novel insights into this understudied topic. Although the analysis is cross-sectional, the use of longitudinal baseline data for adjustment reinforces the examination of the association between entrance area environments and depression. This study has several limitations. First, both the outcome and explanatory variables were based on data collected simultaneously; therefore, causal relationships cannot be established. Second, the analysis was limited to the presence or absence of features such as plants and flowers, without examining the effect of their quantity or quality. Third, the study was conducted in a specific urban area, which may limit the generalizability of the findings. Fourth, residents may have also engaged in gardening in gardens or on balconies, which could have influenced depressive symptoms. Therefore, the present findings may partly reflect the effects of gardening activities conducted in areas other than entrances. Finally, the mechanisms suggested in the discussion linking entrance-area gardening and depressive symptoms remain unclear, and the observed association may be influenced by unmeasured confounding. Further research is needed to clarify these relationships.

## 5 Conclusions

The presence of plants or flowers in entrance areas was associated with a lower prevalence of depression among older adults, highlighting the potential mental health benefits of such features. These findings underscore the importance of considering residential entrance features to support the mental health of older adults. Specifically, in apartment buildings, implementing management policies that allow the placement of plants or flowers near entrances and designing features such as porches or alcoves (recessed spaces in front of doors along common corridors) could provide safe, accessible spaces for greenery unless obstructing evacuation routes. Such measures can help maintain and improve residents' mental health. Future studies should use longitudinal designs and mediation analyses to extensively investigate the causal relationship and underlying mechanisms between entrance area environments and depression.

## CRediT authorship contribution statement

**Hiroaki Yoshida:** Writing – original draft, Visualization, Methodology, Investigation, Formal analysis, Data curation, Conceptualization. **Masamichi Hanazato:** Writing – review & editing, Supervision, Project administration, Methodology, Funding acquisition, Conceptualization. **Yoko Matsuoka:** Writing – review & editing, Methodology, Investigation, Data curation, Conceptualization. **Yu-Ru Chen:** Writing – review & editing, Methodology, Investigation, Data curation, Conceptualization. **Aiko Eguchi:** Writing – review & editing, Validation, Methodology, Investigation, Conceptualization. **Yusuke Mizuno:** Writing – review & editing, Methodology, Investigation. **Hiroki Suzuki:** Writing – review & editing, Supervision.

## Declaration of generative AI and AI-assisted technologies in the writing process

During the preparation of this work, the authors used ChatGPT 4 to improve language and readability. After using this tool, the authors reviewed and edited the content as needed, and take full responsibility for the content of the publication.

## Funding sources

This work was supported by grants from the 10.13039/501100001691Japan Society for the Promotion of Science (KAKENHI 22K04450, 23K16349, 24K17914, and 25K01387); and the Project for Enhancing the Environment to Create Innovation in Regional Core Universities from the Cabinet Office, Government of Japan. Neither the Japan Society for the Promotion of Science nor Cabinet Office or the Government of Japan had any role in the study design, data analysis, interpretation, or the decision to submit the manuscript.

## Declaration of competing interest

The authors declare that they have no known competing financial interests or personal relationships that could have appeared to influence the work reported in this paper.

## Data Availability

Data sharing is not generally available. Data can only be made available through mutual agreement between the authors, the ward office, and the data requesters.

## Appendix A Supplementary data

Fig. S1. Streetscapes in the residential areas of older adult participants in Japan (2022–2023). Fig. S2. Entrance areas (common corridors) of apartment buildings in the residential areas of older adult participants in Japan (2022–2023). Fig. S3. Entrance areas of detached houses in the residential areas of older adult participants in Japan (2022–2023). Image 1
