# Supplementary material for: Association between home entrance characteristics and depression: A cross-sectional study of community-dwelling older adults in Japan
Source: Prev Med Rep. 2025 Jun 20;56:103148. doi: 10.1016/j.pmedr.2025.103148 (PMC12246930; doi:10.1016/j.pmedr.2025.103148)
Supplement: Supplementary file 1 — Fig. S1. Streetscapes in the residential areas of older adult participants in Japan (2022–2023). Fig. S2. Entrance areas (common corridors) of apartment buildings in the residential areas of older adult participants in Japan (2022–2023). Fig. S3. Entrance areas of detached houses in the residential areas of older adult participants in Japan (2022–2023). [file mmc1.docx]

**Association between home entrance characteristics and depression: A cross-sectional study of community-dwelling older adults in Japan**

Supplementary materials

| A  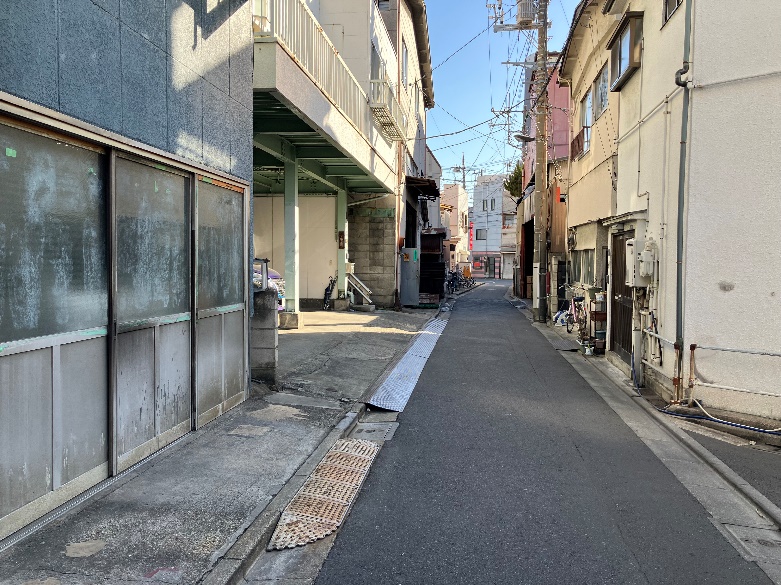 | B  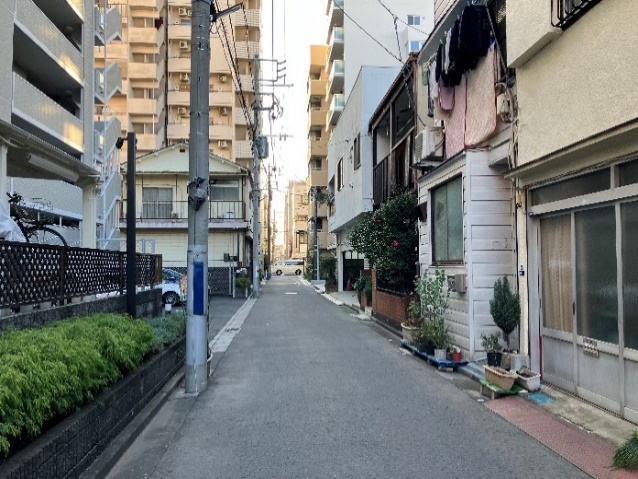 |
| --- | --- |
| Fig. S1. Streetscapes in the residential areas of older adult participants in Japan (2022–2023). (A) Area with densely packed wooden houses. (B) Area with mid- to high-rise condominiums and detached homes. | |

| A  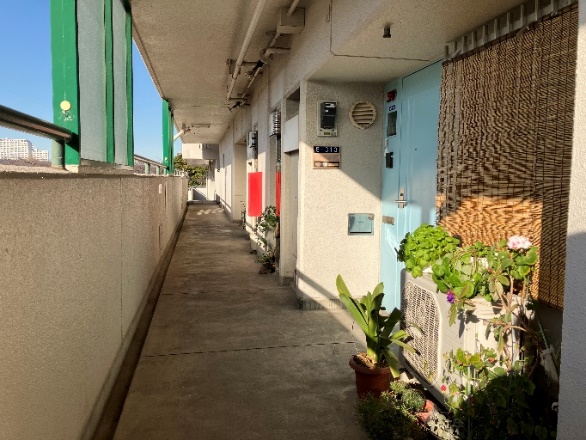 | B  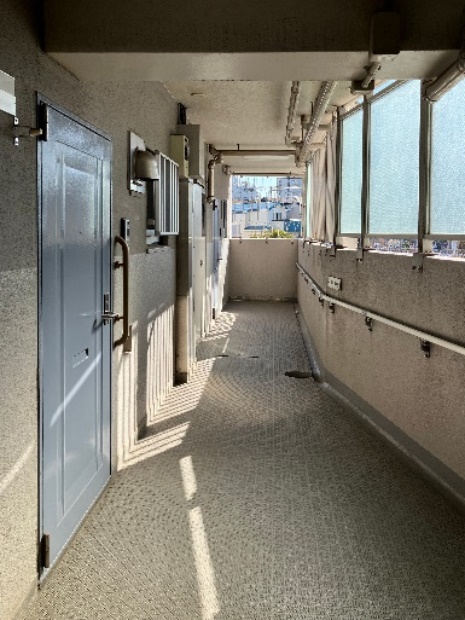 |
| --- | --- |
| Fig. S2. Entrance areas (common corridors) of apartment buildings in the residential areas of older adult participants in Japan (2022–2023). (A) Apartment building with plants or flowers near the entrance. (B) Apartment building with no plants or flowers near the entrance. | |

| A  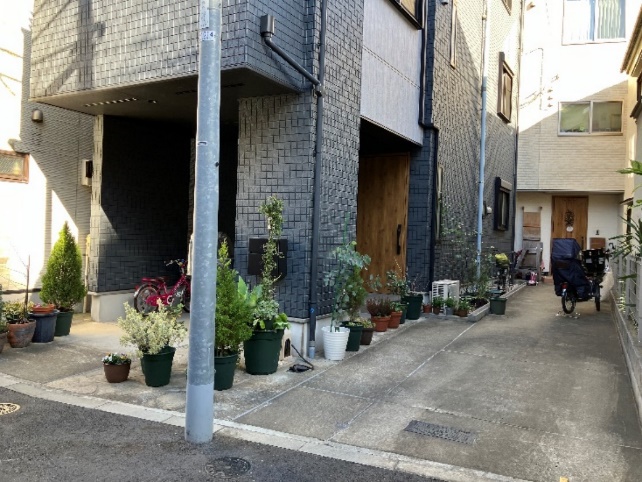 | B  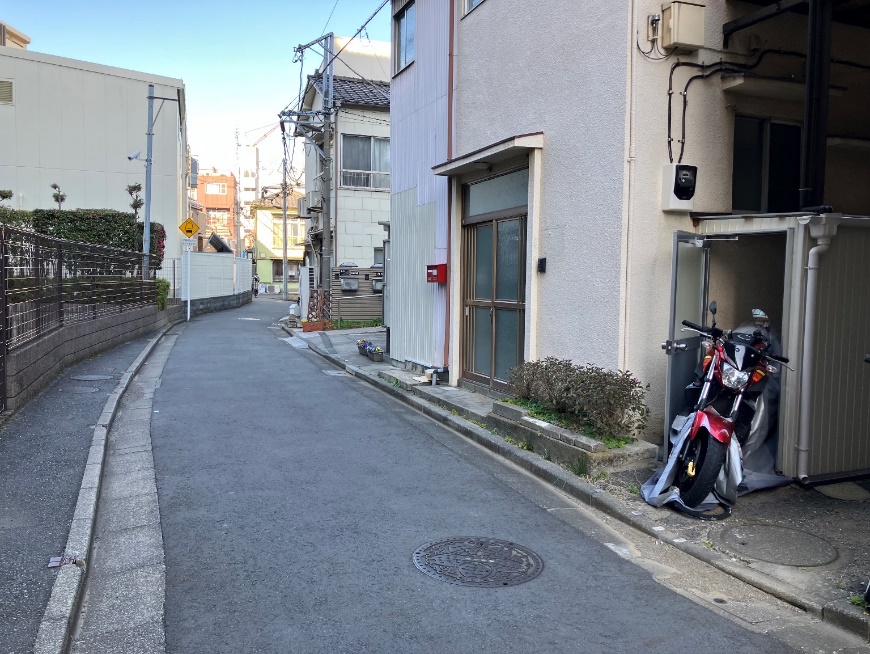 |
| --- | --- |
| Fig. S3. Entrance areas of detached houses in the residential areas of older adult participants in Japan (2022–2023). (A) Houses with many plants and flowers. (B) Houses with few plants planted directly in the ground. | |
